# Cost analysis of lurasidone for the treatment of schizophrenia in adolescents and adults within the United Kingdom

**DOI:** 10.1186/s12913-022-08436-x

**Published:** 2022-08-25

**Authors:** Amy Dymond, Daniela Afonso, Will Green

**Affiliations:** York Health Economics Consortium (YHEC), York, UK

**Keywords:** Schizophrenia, Lurasidone, Cost analysis

## Abstract

**Background:**

Schizophrenia is a serious mental health condition characterised by distortions in thought processes, perception, mood, sense of self, and behaviour. Lurasidone, a second-generation atypical antipsychotic, represents an additional treatment option alongside existing antipsychotics for adolescents and adults with schizophrenia. An economic model was developed to evaluate the incremental costs of lurasidone as a first-line treatment option compared to existing antipsychotics.

**Methods:**

A Markov model was developed to estimate the cost impact of lurasidone as a first-line treatment option for both adolescents and adults. The sequence-based model incorporated the following health states: stable (no relapse or discontinuation), discontinuation (due to adverse events or other reasons), and relapse. Data used to determine the movement of patients between health states were obtained from network meta-analyses (NMAs). The time horizon ranged from three to five years (depending on the patient population) and a six-weekly cycle length was used. Unit costs and resource use were reflective of the UK NHS and Personal Social Services and consisted of the following categories: outpatient, adverse events, primary and residential care. Extensive deterministic sensitivity analysis was undertaken to assess the level of uncertainty associated with the base case results.

**Results:**

Lurasidone is demonstrated to be cost-saving as a first-line treatment within the adolescent and adult populations when compared to second-line and third-line respectively. Lurasidone is more expensive in terms of treatment costs, resource use (in the stable health state) and the treatment of adverse events. However, these costs are outweighed by the savings associated with the relapse health state. Lurasidone remains cost-saving when inputs are varied in sensitivity analysis and scenario analysis.

**Conclusions:**

Lurasidone is a cost-saving first-line treatment for schizophrenia for both adolescents and adults.

**Supplementary Information:**

The online version contains supplementary material available at 10.1186/s12913-022-08436-x.

## Background

Schizophrenia is a serious mental health characterised by distortions in thought processes, perception, mood, sense of self, and behaviour [1]. The symptoms of schizophrenia are typically characterised as positive (such as hallucinations, delusions, and movement disorders), negative (such as emotional apathy, lack of motivation and social withdrawal) and cognitive (such as impairment in cognitive functioning reduced attention span and memory problems) [2]. Schizophrenia can have a detrimental effect on personal, social, educational, and occupational functioning and place a heavy burden on parents and carers [3]. Whilst there is no cure for schizophrenia, evidence shows that it can be treated effectively using pharmacological and psychosocial therapies [4]. Most pharmacological interventions target dopamine pathways in the brain and can broadly be classified into typical and atypical antipsychotics [5].

Lurasidone is a second-generation atypical antipsychotic with a license for the treatment of schizophrenia in both adolescents and adults [6]. Lurasidone represents an additional treatment option alongside existing antipsychotics for adolescents and adults with schizophrenia. There are currently no restrictions on treatment lines of schizophrenia therapy within the UK, and the National Institute for Health and Care Excellence (NICE) advise that “the choice of antipsychotic medication should be made by the service user and healthcare professional together” [7–9].

An economic model was developed to demonstrate the cost impact of lurasidone as a first-line treatment option when compared to alternative antipsychotics for the treatment of schizophrenia in both adolescents and adults from the perspective of the NHS and Personal Social Services. It is anticipated that this analysis will be beneficial to physicians when determining the order in which antipsychotics should be prescribed to adolescents and adults with schizophrenia in the United Kingdom.

## Methods

### Patient populations

The economic model generated results for three distinct populations of people with schizophrenia, adolescents aged 13–17 years, adolescents aged 15–17 years, and adults (aged 18 years and over) [6]. It was necessary to model three distinct populations because lurasidone and haloperidol are currently the only second-generation atypical antipsychotics licensed for patients with schizophrenia between the age of 13–15 [6, 10]. The treatment options were extended to aripiprazole or paliperidone once patients reached the age of 15 [11, 12]. Alternative antipsychotics are licensed for patients aged 18 and over (brexpiprazole and cariprazine). Therefore, patients within the adult population were able to receive either lurasidone, brexpiprazole or cariprazine upon entry to the model.

### Model overview

**Fig. 1:**
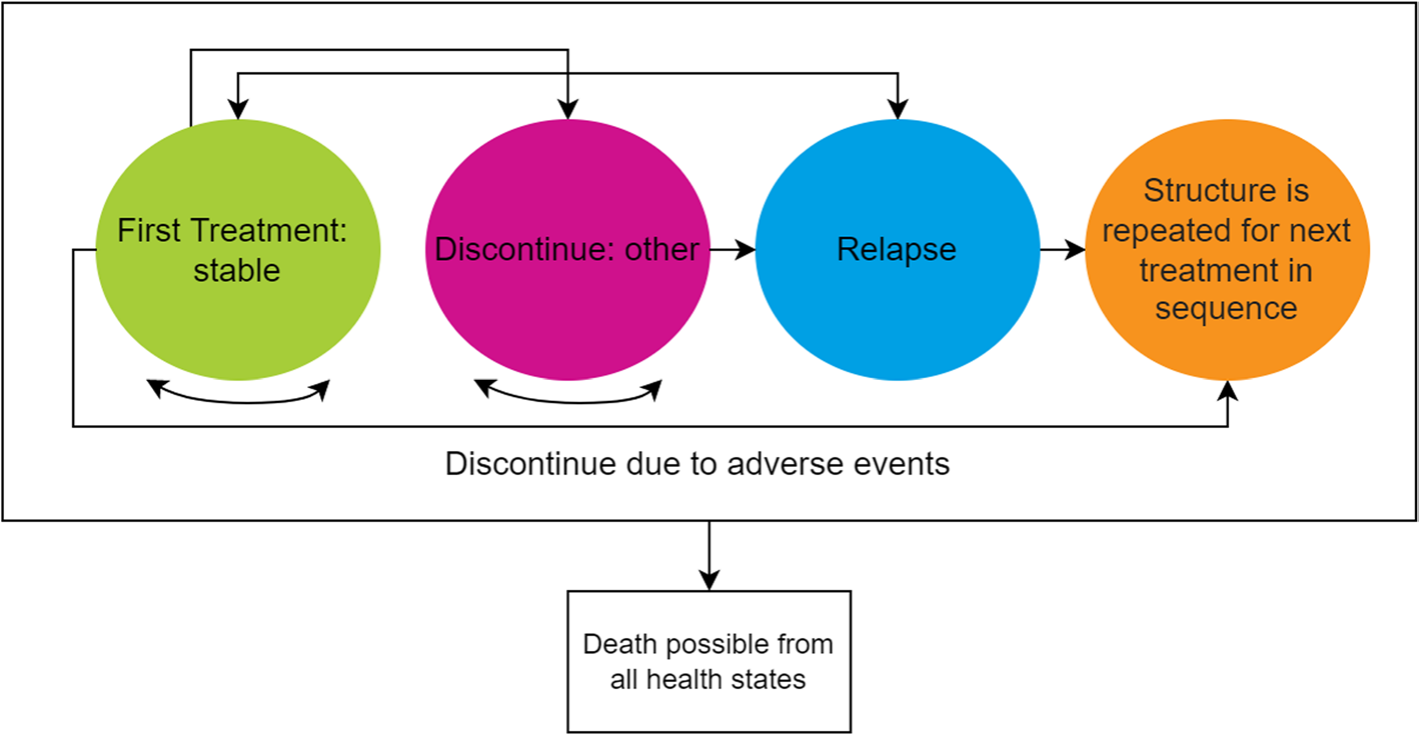
Schematic of base case model structure

A Markov sequenced-based model was developed in Microsoft Excel to assess the cost impact of lurasidone as a first-line treatment when compared to second-line and third-line within the adolescent and adult populations respectively (Fig. 1). The model structure, and corresponding assumptions, was designed in alignment with an economic model developed by NICE to inform the clinical guideline of schizophrenia in adults and a model submitted to NICE for the appraisal of aripiprazole for the treatment of schizophrenia in adolescents aged 15–17 years [13, 14].

A hypothetical cohort with diagnosed schizophrenia entered the model and received first-line treatment with either lurasidone, or an alternative antipsychotic, and their condition was classified as stable (the patient was continuing to receive treatment with no relapse). Following the first six-weekly cycle, patients either:

- Remained in the stable health state;
- Experienced a relapse;
- Discontinued treatment due to intolerable side effects/adverse events;
- Discontinued treatment for any other reasons except relapse or the presence of intolerable side effects;
- Died.

In alignment with the aforementioned NICE models, patients discontinuing first-line treatment due to intolerable side effects/adverse events were assumed to switch to second-line antipsychotic therapy [13, 14]. Patients discontinuing first-line treatment due to other reasons were assumed to stop treatment abruptly and move onto no treatment where they remained until they experienced a relapse. All patients experiencing a relapse were assumed to stop any antipsychotic drug that they had been receiving whilst in the stable health state and, instead, were treated for the acute relapse episode. Following relapse, patients either returned to their previous antipsychotic medication (if they had previously discontinued treatment for other reasons) or switched to second-line treatment. Patients discontinuing treatment because of intolerable side effects, or other reasons, were assumed not to experience a relapse for the remaining duration of the cycle within which discontinuation occurred.

It was assumed that the discontinuation of an antipsychotic because of intolerable side effects only occurred during the first six weeks of use. However, discontinuation due to other reasons was possible across the remainder of cycles within the model. Patients may have also experienced side effects that did not lead to discontinuation (tolerable side effects). In these cases, it was assumed that patients continued to take the antipsychotic until they discontinued it for any other reason. It was also possible for patients to die within any health state.

A six-weekly cycle was chosen to align with the follow-up period included within the network meta-analyses (NMA) that were used to inform treatment discontinuation within the model [15, 16]. All transitions in the model, for purposes of estimations of costs, were assumed to occur in the middle of each cycle. The number of weeks and/or years that a patient spent in each health state was estimated over the time horizon of the model. Time horizons of five and three years were used within the adolescent populations (13–17 years and 15–17 years respectively) to replicate the maximum duration an individual would remain within their initial patient group before being considered an adult. A time horizon of five years was used within the adult population.

The model structure for the adult population was very similar across the adolescent and adult populations. However, the treatment sequences (and subsequent efficacy and safety data), differed across the populations (as presented below). The choice of treatment sequence was determined followed consultation with clinicians. The specific sequences were chosen because they reflected current and expected clinical practice at the time of publication.

- Adolescent (aged 13–17 years and 15–17 years):
- Intervention: Lurasidone, haloperidol, aripiprazole, paliperidone and clozapine vs;
- Comparator: Haloperidol, lurasidone, aripiprazole, paliperidone and clozapine.
- Please note that patients could not receive aripiprazole or paliperidone until they reached the age of 15.
- Adult (aged 18 and over):
- Intervention: Lurasidone, cariprazine, brexpiprazole and clozapine vs;
- Comparator: Cariprazine, brexpiprazole, lurasidone and clozapine.

### Data sources and model parameters

All clinical and safety inputs used to inform the transition of patients throughout the model are presented in Table 1.

**Table 1:** Clinical input data used in model

Antipsychotic	Six-weekly				
	**Discontinuation**		**Relapse**	** ≥ 7% weight gain**	**EPS**
	**Adverse events**	**Other reasons**			
Lurasidone: adolescents (80 mg)	1.3% [15]	8.8% [15]	2.1% [17]	5.9% [18]	28.7% [18]
Haloperidol (3 mg)	8.0% [19]	7.5% [18]	4.9% [20]	9.7% [18]	46.2% [18]
Paliperidone (6 mg)	3.4% [15]	3.8% [15]	1.9% [17]	14.7% [18]	23.8% [18]
Aripiprazole (10 mg)	15.3% [15]	25.7% [15]	4.6% [17]	9.0% [18]	19.5% [18]
Lurasidone: adults (148 mg)	7.6% [16]	18.2% [16]	2.1% [17]	5.9% [18]	28.7% [18]
Brexpiprazole (4 mg)	5.6% [16]	25.6% [16]	2.5% [17]	13.5% [18]	23.7% [18]
Cariprazine (6 mg)	8.5% [16]	28.8% [16]	3.7% [17]	5.6% [18]	32.7% [18]
Clozapine	NA	5.4% [18]	4.9% [21]	33.3% [22]	6.8% [18]
No treatment	NA	NA	7.1% [17]	NA	NA

#### Clinical efficacy

Treatment efficacy was determined by the length of time patients spent within the stable health state throughout the model time horizon.

##### Discontinuation

The six-weekly probabilities of discontinuation due to intolerable adverse events and other reasons associated with the adolescent and adult populations were informed by two separate NMAs [15, 16]. The first NMA was designed to estimate the efficacy and safety of lurasidone, brexpiprazole and cariprazine as treatments for schizophrenia. The systematic review used to inform the feasibility assessment of this NMA only identified one trial within the adolescent population and, therefore, this NMA was limited to the adult population. The second NMA focused on treatments specifically licensed for adolescents (lurasidone, aripiprazole, paliperidone and haloperidol).

The outputs of both NMAs were used to inform the odds ratios of discontinuation, compared to placebo, associated with each of the interventions. Based on these odds ratios, the odds of discontinuation with each intervention could then be estimated when compared with placebo. All odds were then converted to a probability to facilitate inclusion in the economic model. Discontinuation due to a lack of efficacy, as reported in the NMA, was assumed to be a suitable proxy for discontinuation due to other reasons. No studies assessing either the efficacy or the safety of haloperidol were identified from the systematic literature review informing the second NMA. Furthermore, clozapine was not included within either NMA. Therefore, the probabilities of discontinuation associated with haloperidol and clozapine were obtained from alternative sources [18, 19]. Further information regarding the derivation of the six-weekly discontinuation probabilities is presented within the supplementary material (Sect. 1.2).

##### Relapse

The probability of relapse was dependent on the latest antipsychotic treatment received and identified from a targeted literature search. As relapse rates were not considered within the NMAs used to inform the discontinuation parameters described above, the relative risks of relapse associated with the majority of interventions (except haloperidol and clozapine), compared to placebo, were obtained from an alternative NMA that was identified from a targeted literature search [17]. The definition of relapse used within this NMA was dependent on that used in each of the clinical trials. The most common definitions of relapse were a rating scale-based criteria, hospital admission or a combination of the two. The annual relapse rate associated with placebo was converted to a six-weekly probability to account for the cycle length within the model [23]. The relative risk of each antipsychotic, taken from the NMA, was then used to estimate the six-weekly probability of relapse associated with each active intervention.

The relative risks of relapse associated with haloperidol and clozapine were obtained from alternative sources because they were not included within the NMA previously described [17, 20]. The relapse rate associated with no treatment was assumed to be equal to placebo. Further information regarding the estimation of relapse rates used within the model is provided within the supplementary material (Sect. 1.3).

##### Adverse Events

The model incorporated one-off treatment costs for two adverse events that are not necessarily intolerable nor lead patients to discontinue treatment: weight gain of ≥ 7% and extrapyramidal symptoms (EPS). These adverse events were assumed to occur within the first six weeks of treatment and, therefore, one-off treatment costs were applied within the base case analysis.

An NMA of randomised controlled trials of 32 antipsychotics was used to determine the relative risk of a patient experiencing a ≥ 7% increase in weight compared to placebo, except for clozapine which was not reported [18]. The probability of a ≥ 7% weight gain associated with clozapine was obtained from an NHS research study which reported that a third of patients’ experienced ≥ 7% weight gain over three years [22]. This NMA was also used to determine the relative risk of a patient requiring antiparkinsonian medication when taking each antipsychotic compared to placebo (the use of antiparkinsonian medication was used as a proxy for the requirement of treatment for EPS symptoms) [18]. Further information regarding the adverse event inputs used within the model is provided within the supplementary material (Sect. 1.4).

##### Mortality

An all-cause mortality risk, sourced from UK age-related population norms, was applied to all patients when alive within the model [24]. However, there is evidence that the risk of death associated with schizophrenia is greater than the general population. In particular, the authors of a systematic review concluded that the mean standardised mortality ratio (SMR) associated with schizophrenia was 2.6 for all populations [25]. Therefore, this SMR was applied to the age-related population norm mortality data to generate schizophrenia-specific values suitable for the model. It was assumed that the risk of death was independent of specific antipsychotic drug use, or health state, owing to a lack of sufficient data to support an alternative hypothesis.

#### Costs

Four types of costs were captured in the model: treatment acquisition, medication switching, health state-specific and adverse event management (Table 2).

**Table 2:** Cost inputs used in model

**Drug**	**Price per pack**	**Packaging**	**Daily cost**
Lurasidone (adolescents) (80 mg)	£90.72 [26]	28 × 74 mg tablets	£3.50
Lurasidone (adults) (148 mg)	£90.72 [26]	28 × 74 mg tablets	£6.48
Haloperidol (3 mg)	£4.48 [27]	5 mg/5 ml 100 ml solution	£0.13
Paliperidone (6 mg)	£97.28 [28]	28 × 6 mg tablets	£3.47
Aripiprazole (10 mg)	£0.59 [27]	28 × 5 mg tablets	£0.04
Brexpiprazole (4 mg)^a^	£104.47	28 × 6 mg tablets	£2.49
Cariprazine (6 mg)	£80.36 [29]	28 × 6 mg tablets	£2.87
Clozapine (325 mg)	£6.32 [27]	84 × 25 mg tablets	£0.98
**Item**		**Total six-weekly costs (per patient)**	
		**Stable**	**Relapse**
Outpatient, primary, community care and residential care (adults)		£2,000 [3, 13, 27, 30–33]	£27,906 [3, 13, 27, 30–33]
Outpatient, primary, community care and residential care (adolescents)		£701 [3, 13, 27, 30–33]	£28,692 [3, 13, 27, 30–33]
**Item**		**Weight Gain**	**Acute EPS**
Total cost per adverse event		£92 [3, 14, 27, 30–33]	£220 [13, 27, 30–33]

^a^ Assumed to be 30% more expensive than cariprazine (assumption based on current pricing data provided by Angelini on Czech Republic, Denmark, Finland, Italy, Norway, and Slovenia)

##### Treatment acquisition

The unit costs for haloperidol, aripiprazole and clozapine were obtained from NHS EMIT, which provides prices for generic drugs in England [27]. Unit costs for all other treatments were sourced from the BNF except for brexpiprazole. The unit cost of brexpiprazole is not yet publicly available and was assumed to be 30% greater than cariprazine. Patient monitoring costs were also applied to patients receiving clozapine because the prescribing physician must register themselves, a nominated pharmacist, and the patient with the Clozaril Patient Monitoring Service, as outlined in the Summary of Product Characteristics [34]. These requirements equate to monitoring costs associated with approximately three blood tests per six-weekly cycle.

##### Medication switching

Patients moving to the next line of treatment (due to relapse or discontinuation) or returning to the same treatment, following at least one cycle receiving no treatment, incurred costs associated with three visits to a consultant psychiatrist [35].

##### Health state

It was assumed that adult patients within the stable health state either resided within a private household, residential care (sheltered or group), or long-term hospital care (77, 18, 2 and 3% respectively) [13]. Adolescent patients within the stable health state were assumed to reside in a private household only and, therefore, did not incur any residential costs.

Patients experiencing relapse were assumed to receive inpatient treatment within an acute hospital or remain at home whilst receiving support through mental health services (a distribution of 77.30 and 22.70% respectively) [10]. It was assumed that adolescents and adults receiving treatment at home received support from the crisis resolution team for adults with mental health problems and child & adolescent mental health services (CAMHS) respectively. It was also assumed that patients stopped taking their previous antipsychotic medication while in the relapse health state, and instead, receive olanzapine at a dose of 10 mg/day [30]. Six-weekly outpatient, primary and community care costs were also applied to patients within the stable and relapse health states (supplementary tables 7, 8 and 9).

##### Adverse event

All adverse events were assumed to present within the first six weeks and, therefore, these costs were applied upfront within the model (within the first cycle that a patient received each antipsychotic). All patients experiencing ≥ 7% weight gain were assumed to require two visits to their GP for general advice. In addition, 20% of patients received special advice from a dietician (two visits required). It was assumed that all patients requiring treatment for EPS would attend an additional psychiatrist outpatient appointment and receive procyclidine for three months [27, 35]. It was assumed that the resource use associated with each adverse event did not differ by patient age (i.e., it was the same for both adolescents and adults).

All unit costs in the model were inflated to the 2019/20 price year using the most recent Pay and Prices Index within the Personal Social Services Research Unit [31] and are presented in Table 2. Further details associated with the elicitation of these costs is presented with the supplementary material (supplementary table 10).

### Sensitivity analysis

Deterministic sensitivity analysis (DSA) was conducted to account for first-order uncertainty around all clinical effectiveness, cost, and adverse event parameters. Parameters were varied using associated 95% credible intervals where available. All other parameters were varied by ± 20%.

Probabilistic sensitivity analysis (PSA) was undertaken with 1,000 model simulations. Log-normal distributions were fitted to the relative risks and/or odds ratios used to inform the probability of discontinuation and adverse events. Gamma distributions were fitted to the disutility and resource use parameters. In the absence of data on the variability around the sampling distribution of mean values, the standard error was assumed to be equal to 25% of the mean.

## Results

The results presented in Table 3 and 4 are based upon the following treatment sequences:

**Table 3:** Base case results (per patient)

**Total cost (five-years)**	**Intervention sequence**	**Comparator sequence**	**Difference**
Adolescent (13–17 years)	£100,288	£103,375	-£3,088
**Total cost (three-years)**	**Intervention sequence**	**Comparator sequence**	**Difference**
Adolescent (15–17 years)	£52,274	£54,214	-£1,939
**Total cost (five-years)**	**Intervention sequence**	**Comparator sequence**	**Difference**
Adults (18 years and over)	£141,558	£142,355	-£797

**Table 4:** Number of relapses (per patient)

**Total number of relapses (five-years)**	**Intervention sequence**	**Comparator sequence**	**Difference**
Adolescent (13–17 years)	2.669	2.781	-0.112
**Total number of relapses (three-years)**	**Intervention sequence**	**Comparator sequence**	**Difference**
Adolescent (15–17 years)	1.214	1.297	-0.082
**Total number of relapses (five-years)**	**Intervention sequence**	**Comparator sequence**	**Difference**
Adults (18 years and over)	2.407	2.456	-0.049

- Adolescent (aged 13–17 years and 15–17 years):
- Intervention: Lurasidone, haloperidol, aripiprazole, paliperidone and clozapine vs;
- Comparator: Haloperidol, lurasidone, aripiprazole, paliperidone and clozapine.
- Please note that patients could not receive aripiprazole or paliperidone until they reached the age of 15.
- Adult (aged 18 and over):
- Intervention: Lurasidone, cariprazine, brexpiprazole and clozapine vs;
- Comparator: Cariprazine, brexpiprazole, lurasidone and clozapine.

The three-year and five-year base case results demonstrate that lurasidone is cost-saving when used as a first-line treatment, compared to second-line and third-line, within the adult and adolescent populations respectively. Lurasidone as a first-line treatment is more expensive in terms of treatment costs and resource use in the stable health state in all populations. However, lurasidone is associated with a lower number of relapses in all populations. Therefore, these increased costs are outweighed by the savings associated with the relapse health state meaning lurasidone is cost-saving as a first-line treatment when compared to lurasidone as a third-line treatment. A further cost breakdown by resource category is presented within the supplementary appendix (supplementary Sect. 1.5.6).

### Sensitivity analysis

The results of the DSA (as presented within the supplementary appendix) show that the discontinuation rate (due to other reasons) associated with lurasidone is the primary driver of the cost model results when all other inputs remain constant at base case settings. Discontinuation rates (due to intolerable adverse events and other reasons), relapse and residential costs associated with relapse are also key drivers of the model results. Only the discontinuation rate due to other reasons associated with lurasidone caused lurasidone to become cost-incurring as a first-line treatment when compared to second-line, in the adolescent (aged 13–17 population). No other inputs cause lurasidone to become cost-incurring when varied across the three populations. Tornado plots for each of the populations are presented within the supplementary material (see supplementary Figs. 1, 2 and 3).

The results of the PSA are similar to the base case analysis in all populations, with lurasidone as a first-line treatment being cost-saving in all populations (see supplementary Figs. 4, 5 and 6). Lurasidone as a starting treatment was cost-saving in 86.5%, 76.8% and 70.4% of iterations within the 13–17, 13–15 and 18 years and above populations respectively.

## Discussion

The results of the cost model indicate that lurasidone is cost-saving as a first-line treatment for schizophrenia versus second-line, and third-line, within both the adolescent (aged 13–17 and 15–17 years) and adult populations respectively. Lurasidone is associated with greater treatment, stable health state and adverse event costs when used as a first-line treatment within all populations. However, these additional costs are outweighed by the cost-savings incurred through patients spending less time within the relapse health state. Therefore, there could be savings to the NHS for the optimisation of schizophrenia treatment sequences, namely through the use of lurasidone earlier in the sequence.

The model only considers the cost implications of the use of lurasidone as first-line treatment compared to second and third-line (within the adolescent and adult populations respectively), with no consideration of the patient health-related quality of life (which considers the impact that a disease has on a patient’s functioning and well-being). However, it is expected that patients would experience a reduction in quality of life whilst receiving treatment for a relapse episode. Lurasidone is associated with a lower relapse rate and, therefore, it is expected that lurasidone may also lead to a greater quality of life when taken as first-line rather than second and third-line within the adolescent and adult populations, respectively. The increased quality of life associated with lurasidone as a first-line treatment should be demonstrated through further research.

The authors are aware of one previous economic evaluation estimating the cost-utility of lurasidone compared to aripiprazole (both followed by amisulpride and clozapine) in adults with schizophrenia [36]. The results of this economic evaluation are not directly comparable to this study, because it was conducted from a Scottish perspective, and compared different treatment options. However, although the treatment sequence containing lurasidone was associated with higher drug, outpatient and residential costs across both the present and Scottish models, these additional costs were outweighed by savings associated with relapse (which contributed to the greatest proportion of costs).

### Strengths and limitations

The incremental cost of several different treatment sequences could be assessed using the model and several assumptions were made regarding the treatment sequences modelled. The NICE guidelines do not outline any specific treatment sequences and, as such, the sequences modelled may not reflect the order of antipsychotics received by all patients with schizophrenia within the UK. Furthermore, whilst there are similar modes of action between comparators, it is unclear whether the efficacy of subsequent lines of treatment is independent of treatments received previously. Limited evidence was available regarding the potential degradation in treatment effects if a patient had taken an alternative treatment previously. Due to a lack of alternative information to suggest otherwise, the model currently assumes the efficacy of each intervention is not dependent upon the treatment line.

One strength of this model is that the implementation of a Markov model allows the long-term costs of schizophrenia to be estimated and allows sufficient time for patients to receive all treatments within the sequence. The model structure within the adolescent (aged 13–17 years) population also takes into consideration the fact that treatment options change once patients reach the age of 15, due to licencing differences. The structure of the model, and its inputs, also align with an economic model developed by NICE to inform the clinical guideline of schizophrenia in adults and a model submitted to NICE for the appraisal of aripiprazole (TA213) for the treatment of schizophrenia in adolescents aged 15–17 years [13, 14].

The probability of discontinuation due to intolerable adverse events and other reasons, relapse rates and probability of intolerable adverse events were informed by NMAs [15, 16, 18, 37–39]. NMAs are robust sources of efficacy data, since they use both direct and indirect evidence to estimate the comparative efficacy of several interventions to each other and reduce bias caused by differences in trial designs and populations [40]. Therefore, the efficacy data used to inform the model is likely to be particularly robust. However, it was not possible to identify one NMA that reported all the outcomes required to populate the model.

It should be noted that the NMAs used to inform the discontinuation parameters produced results for individual dosages of each antipsychotic, rather than as a combination. However, a wide range of dosages for each intervention are used throughout the UK (the rates for each dose are uncertain). Whilst efficacy data were applied in the model for the doses that are expected to be the most commonly used (according to the BNF and summary of product characteristics), the discontinuation efficacy parameters are unlikely to be reflective of the whole patient population within the UK receiving each antipsychotic given the variation in the doses administered.

The six-weekly probability discontinuation of haloperidol due to intolerable adverse events was also taken from an NMA analysis of the dose–response effects of lurasidone on acute schizophrenia [19]. This NMA reported results of the opposite direction for all dosages of lurasidone, which may positively bias the adolescent results in favour of lurasidone. It must also be noted that the confidence intervals were extremely wide across all NMA results and, therefore, these inputs are associated with uncertainty.

## Conclusions

Lurasidone is demonstrated to be cost-saving when used earlier in the schizophrenia treatment sequence, within all populations, because patients receiving this treatment spend less time within the relapse health state (which is the most-costly health state).

## Supplementary Information

**Additional file 1.** Supplementary materials.

## Data Availability

All data generated or analysed during this study are included in this published article [and its supplementary information file].

## Abbreviations

BNF: British National Formulary
CAMHS: Child and Adolescent Mental Health Services
DSA: Deterministic Sensitivity Analysis
EMIT: Electronic Market Information Tool
EPS: Extrapyramidal Symptoms
GP: General Practitioner
NHS: National Health Service
NICE: National Institute for Health and Care Excellence
NMA: Network Meta-Analysis
PSA: Probabilistic Sensitivity Analysis
SMR: Standardised Mortality Ratio
UK: United Kingdom
YHEC: York Health Economics Consortium
