# Supplementary material for: Cost analysis of lurasidone for the treatment of schizophrenia in adolescents and adults within the United Kingdom
Source: BMC Health Serv Res. 2022 Aug 25;22:1084. doi: 10.1186/s12913-022-08436-x (PMC9404623; doi:10.1186/s12913-022-08436-x)
Supplement: Supplementary file 1 — Additional file 1. Supplementary materials. [file 12913_2022_8436_MOESM1_ESM.docx]

# SUPPLEMENTARY MATERIAL

## Antipsychotic Treatment Dosages

**Table 1. Antipsychotic daily dosages**

| **Antipsychotic** | **Daily dosage (mg)** | **Notes** |
| --- | --- | --- |
| Lurasidone (adolescents) | 80 | Maximum dose assumed [[1](#_ENREF_1)] |
| Haloperidol | 3 | Usual dose 2mg-4mg daily: mid-point assumed [[2](#_ENREF_2)] |
| Paliperidone | 6 | The recommended dose for the treatment of schizophrenia in adults is 6mg once daily [[3](#_ENREF_3), [4](#_ENREF_4)] |
| Lurasidone (adults) | 148 | Maximum dose assumed [[1](#_ENREF_1)] |
| Aripiprazole | 10 | The recommended dose for aripiprazole is 10mg/day administered on a once-a-day schedule [[5](#_ENREF_5)] |
| Brexpiprazole | 4 | Maximum dose assumed [[6](#_ENREF_6)] |
| Cariprazine | 6 | Maximum dose assumed [[7](#_ENREF_7)] |
| Clozapine | 325 | Antipsychotic efficacy can be expected with 200 to 450mg/day - daily dose assumed to be an average [[8](#_ENREF_8)] |

## Discontinuation Inputs

Weighted averages of the six-weekly probability of discontinuation due to any cause, and discontinuation due to adverse events, within both the adult and adolescent populations were first estimated using data from the placebo arm of each study used in the NMAs [[9](#_ENREF_9), [10](#_ENREF_10)], where reported. These calculations estimated six-weekly discontinuation rates associated with placebo of 39.02% & 8.89% due to any cause and intolerable adverse events for the adult population respectively (20.83% & 4.17% within the adolescent populations). The probability of discontinuation due to adverse events was then subtracted from the probability of any cause to prevent double counting. Therefore, the six-weekly probabilities of discontinuation due to any cause and adverse events associated with placebo were 30.13% & 8.89% for the adult population respectively (16.67% & 4.17% within the adolescent populations). These probabilities were then converted to odds of discontinuation (0.431 & 0.098 and 0.200 & 0.043 within the adult and adolescent populations respectively) by dividing the number of patients that discontinued by the number of patients that did not in the respective RCT’s.

The odds ratios estimated for each comparator were then applied to the odds produced for placebo to generate the odds of discontinuation associated with each comparator (as presented in Table 2). Finally, all odds values were then converted to a probability, based on the equation below, with these values presented in Table 3. All discontinuation inputs were varied by the 95% credible intervals within deterministic sensitivity analysis to determine the impact they had on the economic results when varied.

$$Probability= \frac{Odds}{(1+Odds)}$$

The six-weekly probability of discontinuation for other reasons associated with clozapine due to other reasons was obtained from an alternative NMA comparing the safety and efficacy of 32 oral antipsychotics [[11](#_ENREF_11)]. Furthermore, it was not necessary for an input informing the probability of discontinuation due to intolerable adverse events associated with clozapine to be included in the model as this transition was not possible because clozapine was always the last antipsychotic within the sequence.

Although haloperidol was included within the systematic literature review criterion used to inform the NMA informing discontinuation within the adolescent populations, none of the included trials investigated the efficacy, or safety, of this treatment. Therefore, the discontinuation rates associated with haloperidol were identified from a separate targeted literature review. The six-weekly probability of discontinuation with haloperidol due to intolerable adverse events was obtained from an NMA analysing the dose-response effects of lurasidone on acute schizophrenia [[12](#_ENREF_12)]. The NMA reported a relative risk of discontinuation due to adverse events for placebo compared to haloperidol of 0.52 (95% credible intervals: 0.24 to 1.09). This relative risk was divided by the six-weekly probability of discontinuation due to adverse events associated with placebo (4.17%) to estimate the six-weekly probability of discontinuation associated with haloperidol (8%; 95% credible intervals: 3.8% to 17.4%).

The six-weekly probability discontinuation due to other reasons associated with haloperidol was also obtained from the NMA comparing the safety and efficacy of 32 oral antipsychotics [[11](#_ENREF_11)]. The NMA reported the relative risks of discontinuation due to lack of efficacy were 0.45 compared to placebo (95% credible intervals: 0.38 to 0.53), which was used to estimate a six-weekly discontinuation probability of 7.5% using the methods described above (95% credible intervals: 6.3% to 8.8%).

**Table 2. Odds ratios for discontinuation due to adverse events and any other reasons compared with placebo**

| **Drug** | **Odds ratios of discontinuation due to adverse events** | | | **Odds ratios of discontinuation due to other reasons** | | |
| --- | --- | --- | --- | --- | --- | --- |
|  | **Mean** | **Lower Cl** | **Upper Cl** | **Mean** | **Lower Cl** | **Upper Cl** |
| Lurasidone (adolescents) (80mg) | 3.226 [[10](#_ENREF_10)] | 0.885 | 16.667 | 2.083 [[10](#_ENREF_10)] | 0.943 | 5.000 |
| Haloperidol* (3mg) | 0.520 [[12](#_ENREF_12)] | 0.240 | 1.090 | 2.222 [[11](#_ENREF_11)] | 1.887 | 2.632 |
| Paliperidone (6mg) | 1.250 [[10](#_ENREF_10)] | 0.565 | 2.778 | 5.000 [[10](#_ENREF_10)] | 2.000 | 14.286 |
| Aripiprazole (10mg) | 0.240 [[10](#_ENREF_10)] | 0.032 | 1.075 | 0.578 [[10](#_ENREF_10)] | 0.239 | 1.333 |
| Lurasidone: adults (148mg) | 1.189 [[9](#_ENREF_9)] | 0.374 | 4.601 | 1.943 [[9](#_ENREF_9)] | 1.167 | 3.288 |
| Brexpiprazole (4mg) | 1.634 [[9](#_ENREF_9)] | 1.070 | 2.479 | 1.254 [[9](#_ENREF_9)] | 0.929 | 1.630 |
| Cariprazine (6mg) | 1.046 [[9](#_ENREF_9)] | 0.518 | 2.153 | 1.068 [[9](#_ENREF_9)] | 0.674 | 1.758 |
| Clozapine | NA | NA | NA | 0.180 [[11](#_ENREF_11)] | 0.09 | 0.31 |

Cl: Confidence interval; *Applied as a relative risk.

Note: An odds or relative risk <1 favours placebo (the probability of discontinuation will be lower for placebo than the relevant treatment).

**Table 3. Six-weekly discontinuation probabilities due to adverse events and any other reasons vs placebo**

| **Drug** | **Probability of discontinuation due to adverse events** | | | **Probability of discontinuation due to other reasons** | | |
| --- | --- | --- | --- | --- | --- | --- |
|  | **Mean** | **Lower Cl** | **Upper Cl** | **Mean** | **Lower Cl** | **Upper Cl** |
| Lurasidone (adolescents) (80mg) | 1.3% | 0.3% | 4.7% | 8.8% | 3.8% | 17.5% |
| Haloperidol (3mg) | 8.0% | 3.8% | 17.4% | 7.5% | 6.3% | 8.8% |
| Paliperidone (6mg) | 3.4% | 1.5% | 7.1% | 3.8% | 1.4% | 9.1% |
| Aripiprazole (10mg) | 15.3% | 3.9% | 58.0% | 25.7% | 13.0% | 45.6% |
| Lurasidone (adults) (148mg) | 7.6% | 2.1% | 20.7% | 18.2% | 11.6% | 27.0% |
| Brexpiprazole (4mg) | 5.6% | 3.8% | 8.4% | 25.6% | 20.9% | 31.7% |
| Cariprazine (6mg) | 8.5% | 4.3% | 15.8% | 28.8% | 19.7% | 39.0% |

Cl: Confidence interval

Note: An odds or relative risk <1 favours placebo (the probability of discontinuation will be lower for placebo than the relevant treatment).

## Relapse Inputs

The relative risks were informed from an NMA that centred on the results from randomised controlled trials [[13](#_ENREF_13)]. The annual relapse rate associated with placebo (64.2%), reported within the study from which the relative risks associated with the majority of alternative interventions were obtained, was converted to a six-weekly probability to account for the cycle length within the model (7.1%), using the following formula [[14](#_ENREF_14)]:

$$Probability= 1-{exp}^{-rt}$$

Within this formula ‘r’ represented the relapse rate and ‘t’ represented the time point of reference (six weeks). The relative risk of each comparator was then used to estimate the six-weekly probability of relapse associated with each active intervention.

**Table 4. Relative risk of relapse of active intervention vs placebo**

| **Drug** | **Relative relapse rate vs placebo** | **Six-weekly relapse rate vs placebo** |
| --- | --- | --- |
| Lurasidone | 3.33 [[15](#_ENREF_15)] | 2.1% |
| Haloperidol | 1.45 [[16](#_ENREF_16)] | 4.9% |
| Paliperidone | 3.85 [[15](#_ENREF_15)] | 1.9% |
| Aripiprazole | 1.56 [[15](#_ENREF_15)] | 4.6% |
| Brexpiprazole | 2.86 [[15](#_ENREF_15)] | 2.5% |
| Cariprazine | 1.92 [[15](#_ENREF_15)] | 3.7% |
| Clozapine | 1.47 [[17](#_ENREF_17)] | 4.9% |
| No treatment | 1.00 [[15](#_ENREF_15)] | 7.1% |

Note: An odds or relative risk <1 favours placebo (the probability of relapse will be lower for placebo than the relevant treatment.

## Adverse Event Inputs

An NMA of randomised controlled trials of 32 antipsychotics was used to determine the relative risk of a patient experiencing a ≥ 7% increase in weight when taking each antipsychotic compared to placebo and, hence, requiring treatment for all antipsychotics except clozapine (which was not reported) [[11](#_ENREF_11)]. These relative risks were multiplied by the six-weekly probability of a ≥ 7% weight gain associated with placebo (4.8%), which was obtained from a meta-analysis reporting the risk of weight gain associated with olanzapine, paliperidone and risperidone [[18](#_ENREF_18)]. The probability of a ≥ 7% weight gain associated with clozapine was obtained from an NHS research study which stated that a third of patients experienced ≥ 7% weight gain over three years [[19](#_ENREF_19)]. In alignment with the assumption that adverse events occur soon after treatment commencement, it was assumed that patients receiving treatment with clozapine would have experienced this weight gain within the first six weeks of treatment, due to the absence of alternative information.

The use of antiparkinsonian medication was used as a proxy for the requirement of treatment for EPS symptoms. The NMA of randomised controlled trials of 32 antipsychotics was also used to determine the relative risk of a patient requiring antiparkinsonian medication when taking each antipsychotic compared to placebo [[11](#_ENREF_11)]. These relative risks were multiplied by the probability of EPS associated with placebo (14.8%), which was informed from an alternative meta-analysis that also reported the use of antiparkinsonian medication, assuming that EPS symptoms occurred within the first six weeks of treatment [[20](#_ENREF_20)].

The six-weekly probability of each adverse event, associated with each antipsychotic, is presented in Table 5**.**

**Table 5. Relative risk ≥ 7% weight gain and EPS compared to placebo**

|  | **≥ 7%** **weight gain** | | **EPS** | |
| --- | --- | --- | --- | --- |
| **Drug** | **Relative risk compared to placebo** | **Six-weekly probability** | **Relative risk compared to placebo** | **Six-weekly probability** |
| Lurasidone | 1.23 [[11](#_ENREF_11)] | 5.9% | 1.94 [[11](#_ENREF_11)] | 28.7% |
| Haloperidol | 2.02 [[11](#_ENREF_11)] | 9.7% | 3.12 [[11](#_ENREF_11)] | 46.2% |
| Paliperidone | 3.05 [[11](#_ENREF_11)] | 14.7% | 1.61 [[11](#_ENREF_11)] | 23.8% |
| Aripiprazole | 1.86 [[11](#_ENREF_11)] | 9.0% | 1.32 [[11](#_ENREF_11)] | 19.5% |
| Brexpiprazole | 2.81 [[11](#_ENREF_11)] | 13.5% | 1.60 [[11](#_ENREF_11)] | 23.7% |
| Cariprazine | 1.16 [[11](#_ENREF_11)] | 5.6% | 2.21 [[11](#_ENREF_11)] | 32.7% |
| Clozapine | N/A | 33.3% | 0.46 [[11](#_ENREF_11)] | 6.8% |
| Note: A relative risk >1 favours placebo (the probability of ≥ 7% weight gain and EPS symptoms will be lower for placebo than the relevant treatment) | | | | |

## Cost Inputs

### Treatment costs

**Table 6. Treatment-Specific Costs for Schizophrenia**

| **Drug** | **Dose per day** | **Price per pack** | **Packaging** | **Daily cost** |
| --- | --- | --- | --- | --- |
| Lurasidone (adolescents) | 80mg | £90.72 | 28 x 74mg tablets | £3.50 |
| Lurasidone (adults) | 148mg | £90.72 | 28 x 74mg tablets | £6.48 |
| Haloperidol | 3mg | £4.48 | 5mg/5ml 100ml solution | £0.13 |
| Paliperidone | 6mg | £97.28 | 28 x 6mg tablets | £3.47 |
| Aripiprazole | 10mg | £0.59 | 28 x 5mg tablets | £0.04 |
| Brexpiprazole* | 4mg | £104.47 | 28 x 6mg tablets | £2.49 |
| Cariprazine | 6mg | £80.36 | 28 x 6mg tablets | £2.87 |
| Clozapine | 325mg | £6.32 | 84 x 25mg tablets | £0.98 |

*Assumed to be 30% more expensive than cariprazine (assumption based on current pricing data provided by Angelini on Czech Republic, Denmark, Finland, Italy, Norway, and Slovenia)

### Outpatient, Primary and Community Care

Data to inform these inputs were taken from a study that collected health care resource use data from 145 patients with schizophrenia in Leicester, UK [[21](#_ENREF_21)]. This source was also used to inform the resource use within the NICE guideline model [[22](#_ENREF_22)]. The frequency of resource use was first converted from six-monthly to six-weekly to align with the cycle length within the model. These six-weekly resource use values were then multiplied by each unit cost to estimate the total cycle cost per patient. Where possible, unit costs were obtained from the most recent Personal Social Services Research Unit (PSSRU) [[23](#_ENREF_23)].

**Table 7. Outpatient, primary and community care costs (per patient)**

| **Resource use category** | **Unit Cost** | **Visits per patient: six-weekly** | | **Total cost per patient: six-weekly** | |
| --- | --- | --- | --- | --- | --- |
|  |  | **Stable** | **Relapse** | **Stable** | **Relapse** |
| Outpatient psychiatric visit | £167 [[22](#_ENREF_22)] | 0.32 [[22](#_ENREF_22)] | 0.48 [[22](#_ENREF_22)] | £53.77 | £80.66 |
| Outpatient other visits | £111[[22](#_ENREF_22)] | 0.02 [[22](#_ENREF_22)] | 0.07 [[22](#_ENREF_22)] | £2.55 | £7.66 |
| Day hospital visits | £111 [[22](#_ENREF_22)] | 0.53 [[22](#_ENREF_22)] | 0.48 [[22](#_ENREF_22)] | £58.72 | £53.61 |
| Community mental health centre visits | £148 [[22](#_ENREF_22)] | 0.55 [[22](#_ENREF_22)] | 0.32 [[22](#_ENREF_22)] | £81.70 | £47.66 |
| Day care centre visits | £111 [[22](#_ENREF_22)] | 1.36 [[22](#_ENREF_22)] | 0.21 [[22](#_ENREF_22)] | £150.63 | £22.98 |
| Group therapy | £111 [[22](#_ENREF_22)] | 0.09 [[22](#_ENREF_22)] | 0.02 [[22](#_ENREF_22)] | £10.21 | £2.55 |
| Sheltered workshop | £58 [[22](#_ENREF_22)] | 0.25 [[22](#_ENREF_22)] | 0.00 [[22](#_ENREF_22)] | £14.67 | £0.00 |
| Specialist education | £111 [[22](#_ENREF_22)] | 0.67 [[22](#_ENREF_22)] | 0.00 [[22](#_ENREF_22)] | £74.04 | £0.00 |
| Other (not specified) | £59 [[22](#_ENREF_22)] | 0.14 [[22](#_ENREF_22)] | 0.00 [[22](#_ENREF_22)] | £8.14 | £0.00 |
| Psychiatrist visits | £279 [[22](#_ENREF_22)] | 0.58 [[22](#_ENREF_22)] | 0.53 [[22](#_ENREF_22)] | £160.43 | £147.59 |
| Psychologist visits | £233 [[22](#_ENREF_22)] | 0.00 [[22](#_ENREF_22)] | 0.00 [[22](#_ENREF_22)] | £0.00 | £0.00 |
| GP visits | £39 [[23](#_ENREF_23)] | 0.41 [[22](#_ENREF_22)] | 0.37 [[22](#_ENREF_22)] | £16.15 | £14.35 |
| District nurse visits | £28 [[23](#_ENREF_23)] | 0.02 [[22](#_ENREF_22)] | 0.00 [[22](#_ENREF_22)] | £0.64 | £0.00 |
| Community psychiatric nurse visits | £23 [[22](#_ENREF_22)] | 2.90 [[22](#_ENREF_22)] | 1.20 [[22](#_ENREF_22)] | £66.10 | £27.27 |
| Social worker visits | £17 [[23](#_ENREF_23)] | 0.02 [[22](#_ENREF_22)] | 0.09 [[22](#_ENREF_22)] | £0.40 | £1.59 |
| Occupational therapist visit | £45 [[23](#_ENREF_23)] | 0.00 [[22](#_ENREF_22)] | 0.18 [[22](#_ENREF_22)] | £0.00 | £8.28 |
| Home healthcare worker | £30 [[23](#_ENREF_23)] | 0.09 [[22](#_ENREF_22)] | 0.14 [[22](#_ENREF_22)] | £2.76 | £4.14 |
| **Total health state cost per patient** | | | | **£700.90** | **£418.34** |

### Relapse

In alignment with the NICE guidelines model, patients experiencing relapse were assumed to require treatment as an inpatient within an acute hospital or remain at home whilst receiving support through mental health services (a distribution of 77.30% and 22.70% respectively) [[22](#_ENREF_22)]. The average cost of hospitalisation per relapse, for both adults and adolescents, was based on a unit cost per bed day (£469) associated with a psychotic crisis [[24](#_ENREF_24)]. This unit cost per bed day was multiplied by the mean length of stay associated with schizophrenia, schizotypal and delusional disorders as reported within Hospital Episode Statistics (75 days), for patients treated within a hospital setting [[25](#_ENREF_25)].

**Table 8. Costs and resource use (relapse)**

| **Item** | **Unit cost per episode** | **% of patients (adults)** | **% of patients (adolescents)** |
| --- | --- | --- | --- |
| Acute hospital (NHS ref code: WD07Z) | £35,175 [[25](#_ENREF_25)] | 77.3% [[22](#_ENREF_22)] | 77.3% [[22](#_ENREF_22)] |
| Mental health services (adults) | £1,296* [[26](#_ENREF_26)] | 22.7% |  |
| Mental health services (adolescents) | £4,757** [[27](#_ENREF_27)] |  | 22.7% |
| Olanzapine (10mg per day) | £3.43 [[28](#_ENREF_28), [29](#_ENREF_29)] | 100% | 100% |
| **Weighted cost per relapse (per patient)** | | **£27,484** | **£28,270** |

*Crisis resolution teams for adults with mental health problems, cost of case per care staff per week, taken from PSSRU (2007) and inflated to 2019/20 price year. Estimated using the average length of an episode: 27 days;

** Generic single-disciplinary CAMHS team – cost per case, taken from PSSRU 2017, inflated to 2019/20 price year.

### Stable

For all non-private residential settings, weekly per-patient costs were first estimated, which were then multiplied by six, in alignment with the six-weekly cycle length within the model. The weekly unit cost per patient associated with sheltered residential care was obtained from the PSSRU (2020) [[23](#_ENREF_23)]. The unit costs associated with group residential and long-term hospital care were obtained from the NICE guidelines model, due to an absence of a more recent cost source and inflated to the 2019/20 price year [[22](#_ENREF_22)].

**Table 9.** **Costs and resource use (stable)**

| **Accommodation type** | **Unit cost (weekly)** | **Six-weekly cost (per patient)** | **Proportion: Adults** | **Proportion: Adolescents** | **Weighted six-weekly cost (adults)** | **Weighted six-weekly cost (adolescents)** |
| --- | --- | --- | --- | --- | --- | --- |
| Private household | £0 | £0 | 77% | 100% | £0 | £0 |
| Residential care (sheltered) | £842 [[30](#_ENREF_30)] | £5,052 | 18% | 0% | £909.36 | £0 |
| Residential care (group) | £136 [[31](#_ENREF_31)] | £816 | 2% | 0% | £16.32 | £0 |
| Long-term hospital care | £2,072 [[32](#_ENREF_32)] | £12,432 | 3% | 0% | £372.96 | £0 |
| **Total weighted residential cost per patient** | | | | | **£1,299** | **£0** |

### Adverse Events

**Table 10. Treatment-related adverse event costs (per patient)**

| **Adverse event** | **Item** | **% of patients** | **Unit cost** | **Weighted treatment cost** |
| --- | --- | --- | --- | --- |
| Weight gain | Two GP visits (general advice) | 100% | £39.00 [[30](#_ENREF_30)] | £78.00 |
|  | Two dietician visits | 20% | £34.00 [[30](#_ENREF_30)] | £13.60 |
| **Total cost per adverse event** | | | | **£91.60** |
| Acute EPS | Procyclidine (2.5mg three times a day for three months) | 100% | £2.40 [[33](#_ENREF_33)] | £8.51 |
|  | One psychiatrist visit | 100% | £211 [[24](#_ENREF_24)] | £211 |
| **Total cost per adverse event** | | | | **£219.51** |

### Results

***Adolescent (13-17 years)***

**Table 11. Cost breakdown (adolescent [13-17 years]): Lurasidone as first-line vs second-line (per patient over five years)**

|  | **Intervention sequence** | **Comparator sequence** | **Difference** |
| --- | --- | --- | --- |
| Treatment cost | £2,879 | £2,717 | £163 |
| Heath state: Stable | £26,804 | £26,722 | £82 |
| Health state: Relapse | £70,424 | £73,770 | -£3,345 |
| Adverse events | £180 | £167 | £13 |
| **Total** | **£100,288** | **£103,375** | **-£3,088** |

**Table 12: Cost breakdown (adolescent [13-17 years]): Intervention treatment sequence (per patient over five years)**

|  | **Lurasidone** | **Haloperidol** | **Aripiprazole** | **Paliperidone** | **Clozapine** | **Total** |
| --- | --- | --- | --- | --- | --- | --- |
| Treatment cost | £1,374 | £578 | £352 | £510 | £66 | **£2,879** |
| Heath state: Stable | £13,149 | £7,704 | £3,537 | £1,993 | £421 | **£26,804** |
| Health state: Relapse | £25,761 | £33,114 | £8,638 | £2,108 | £803 | **£70,424** |
| Adverse events | £34 | £95 | £28 | £19 | £3 | **£180** |
| **Total** | **£40,318** | **£41,492** | **£12,555** | **£4,630** | **£1,293** | **£100,288** |

**Table 13: Cost breakdown (adolescent [13-17 years]): Comparator treatment sequence (per patient over five years)**

|  | **Haloperidol** | **Lurasidone** | **Aripiprazole** | **Paliperidone** | **Clozapine** | **Total** |
| --- | --- | --- | --- | --- | --- | --- |
| Treatment cost | £43 | £1,753 | £349 | £506 | £66 | **£2,717** |
| Heath state: Stable | £9,970 | £10,850 | £3,508 | £1,977 | £418 | **£26,722** |
| Health state: Relapse | £25,083 | £37,232 | £8,565 | £2,091 | £799 | **£73,770** |
| Adverse events | £55 | £61 | £28 | £19 | £3 | **£167** |
| **Total** | **£35,151** | **£49,895** | **£12,451** | **£4,593** | **£1,286** | **£103,375** |

***Adolescent (15-17 years)***

**Table 14. Cost breakdown (adolescent [15-17 years]): Lurasidone as first-line vs second-line (per patient over three years)**

|  | **Intervention sequence** | **Comparator sequence** | **Difference** |
| --- | --- | --- | --- |
| Treatment cost | £2,093 | £1,683 | £410 |
| Heath state: Stable | £17,165 | £17,106 | £59 |
| Health state: Relapse | £32,886 | £35,293 | -£2,407 |
| Adverse events | £130 | £131 | £1 |
| **Total** | **£52,274** | **£54,214** | **-£1,939** |

**Table 15: Cost breakdown (adolescent [15-17 years]): Intervention treatment sequence (per patient over three years)**

|  | **Lurasidone** | **Haloperidol** | **Aripiprazole** | **Paliperidone** | **Clozapine** | **Total** |
| --- | --- | --- | --- | --- | --- | --- |
| Treatment cost | £1,296 | £425 | £192 | £166 | £15 | **£2,093** |
| Heath state: Stable | £11,190 | £3,935 | £1,400 | £563 | £77 | **£17,165** |
| Health state: Relapse | £20,488 | £8,582 | £3,158 | £522 | £136 | **£32,886** |
| Adverse events | £34 | £71 | £16 | £8 | £1 | **£130** |
| **Total** | **£33,008** | **£13,013** | **£4,766** | **£1,259** | **£229** | **£52,274** |

**Table 16: Cost breakdown (adolescent [15-17 years]): Comparator treatment sequence (per patient over three years)**

|  | **Haloperidol** | **Lurasidone** | **Aripiprazole** | **Paliperidone** | **Clozapine** | **Total** |
| --- | --- | --- | --- | --- | --- | --- |
| Treatment cost | £42 | £1,268 | £192 | £166 | £15 | **£1,683** |
| Heath state: Stable | £8,746 | £6,320 | £1,400 | £563 | £77 | **£17,106** |
| Health state: Relapse | £21,464 | £10,013 | £3,158 | £522 | £136 | **£35,293** |
| Adverse events | £55 | £52 | £16 | £8 | £1 | **£131** |
| **Total** | **£30,307** | **£17,653** | **£4,766** | **£1,259** | **£229** | **£54,214** |

***Adults (18 years and over)***

**Table 17.** **Cost breakdown (adults [18 years and over]): Lurasidone as first-line vs third-line (per patient over five years)**

|  | **Intervention sequence** | **Comparator sequence** | **Difference** |
| --- | --- | --- | --- |
| Treatment cost | £3,179 | £2,620 | £559 |
| Heath state: Stable | £76,851 | £76,746 | £105 |
| Health state: Relapse | £61,380 | £62,846 | -£1,466 |
| Adverse events | £148 | £142 | £6 |
| **Total** | **£141,558** | **£142,355** | **-£797** |

**Table 18: Cost breakdown (adults [18 years and over]): Intervention treatment sequence (per patient over five years)**

|  | **Lurasidone** | **Cariprazine** | **Brexpiprazole** | **Clozapine** | **Total** |
| --- | --- | --- | --- | --- | --- |
| Treatment cost | £1,436 | £878 | £563 | £302 | **£3,179** |
| Heath state: Stable | £33,158 | £23,072 | £13,059 | £7,563 | **£76,851** |
| Health state: Relapse | £26,405 | £19,735 | £9,920 | £5,319 | **£61,380** |
| Adverse events | £34 | £65 | £36 | £13 | **£148** |
| **Total** | **£61,033** | **£43,750** | **£23,578** | **£13,196** | **£141,558** |

**Table 19: Cost breakdown (adults [18 years and over]): Comparator treatment sequence (per patient over five years)**

|  | **Cariprazine** | **Brexpiprazole** | **Lurasidone** | **Clozapine** | **Total** |
| --- | --- | --- | --- | --- | --- |
| Treatment cost | £445 | £865 | £1,008 | £302 | **£2,620** |
| Heath state: Stable | £32,457 | £24,156 | £12,572 | £7,562 | **£76,746** |
| Health state: Relapse | £29,501 | £19,346 | £8,680 | £5,319 | **£62,846** |
| Adverse events | £38 | £53 | £38 | £13 | **£142** |
| **Total** | **£62,441** | **£44,420** | **£22,296** | **£13,196** | **£142,355** |

## Sensitivity Analysis

### Deterministic sensitivity analysis

**Supplementary figure 1. Tornado plots of intervention sequence vs comparator sequence (adolescent aged 13-17 years)**

**Supplementary figure 2. Tornado plots of intervention sequence vs comparator sequence (adolescent aged 15-17 years)**

**Supplementary figure 3. Tornado plots of intervention sequence vs comparator sequence (adults aged 18 years and older)**

### Probabilistic sensitivity analysis

**Supplementary figure 4. Distribution of probabilistic incremental costs (adolescent aged 13-17 years)**

**Supplementary figure 5. Distribution of probabilistic incremental costs (adolescent aged 15-17 years)**

**Supplementary figure 6. Distribution of probabilistic incremental costs (adults aged 18 years and older)**

# REFERENCES

1. European Medicines Agency. Latuda. [Internet]. Amsterdam. 2020. [13 October 2020]. Available from: <https://www.ema.europa.eu/en/medicines/human/EPAR/latuda>.

2. National Institute of Health and Care Excellence (NICE). Haloperidol. [Internet]. Available from: <https://bnf.nice.org.uk/drug/haloperidol.html#indicationsAndDoses>.

3. National Institute for Health and Care Excellence (NICE). Paliperidone. [Internet]. Available from: <https://bnf.nice.org.uk/medicinal-forms/paliperidone.html>.

4. Electronic Medicine Compendium (EMC). Invega 3 mg prolonged-release tablets. [Internet]. 2018. Available from: <https://www.medicines.org.uk/emc/product/6816/smpc>.

5. Electronic Medicine Compendium (EMC). Aripiprazole 5mg tablets. [Internet]. 2020. Available from: <https://www.medicines.org.uk/emc/product/7073/smpc>.

6. European Medicines Agency. Brexpiprazole. [Internet]. Available from: <https://www.ema.europa.eu/en/documents/product-information/rxulti-epar-product-information_en.pdf>.

7. Electronic Medicine Compendium (EMC). Cariprazine. [Internet]. Available from: <https://www.ema.europa.eu/en/documents/product-information/reagila-epar-product-information_en.pdf>.

8. Electronic Medicine Compendium (EMC). Clozaril 25mg and 100mg Tablets. [Internet]. 2020. Available from: <https://www.medicines.org.uk/emc/medicine/32564>.

9. Sanderson A, McCool R, Wood H, Bunney M, Phalguni A. Systematic review and feasibility assessment for a network meta-analysis of treatments for schizophrenia [CRD42020167883]. 2020.

10. Watkins D, Wilson K, Wood H, Beale S, Patterson J, Mclean B. Systematic review, feasibility assessment and network meta-analysis of treatments for adolescents with schizophrenia [CRD42021244609]. 2021.

11. Huhn M, Nikolakopoulou A, Schneider-Thoma J, Krause M, Samara M, Peter N, et al. Comparative efficacy and tolerability of 32 oral antipsychotics for the acute treatment of adults with multi-episode schizophrenia: a systematic review and network meta-analysis. The Lancet. 2019;394(10202):939-51.

12. Srisurapanont M, Suttajit S, Likhitsathian S, Maneeton B, Maneeton N. A network meta-analysis of the dose–response effects of lurasidone on acute schizophrenia. Scientific reports. 2021;11(1):1-3.

13. Zhao YJ, Lin L, Teng M, Khoo AL, Soh LB, Furukawa TA, et al. Long-term antipsychotic treatment in schizophrenia: systematic review and network meta-analysis of randomised controlled trials. BJPsych Open. 2016;2(1):59-66.

14. Gidwani R. Deriving Transition Probabilities for Decision Models. 2014. Available from: <https://pdfs.semanticscholar.org/5c71/d24a836b8a88fa83ac9f74d154163bea3e79.pdf>.

15. Kearns B, Cooper K, Cantrell A, Thomas C. Schizophrenia Treatment with Second-Generation Antipsychotics: A Multi-Country Comparison of the Costs of Cardiovascular and Metabolic Adverse Events and Weight Gain. Neuropsychiatric Disease and Treatment. 2021;17:125-37.

16. Adams CE, Bergman H, Irving CB, Lawrie S. Haloperidol versus placebo for schizophrenia. Cochrane Database of Systematic Reviews. 2013(11):CD003082.

17. Kishimoto T, Agarwal V, Kishi T, Leucht S, Kane JM, Correll CU. Relapse prevention in schizophrenia: a systematic review and meta-analysis of second-generation antipsychotics versus first-generation antipsychotics. Molecular psychiatry. 2013;18(1):53-66.

18. Spertus J, Horvitz-Lennon M, Abing H, Normand SL. Risk of weight gain for specific antipsychotic drugs: a meta-analysis. npj Schizophrenia. 2018;4(1):12.

19. Health Research Authority (HRA). Why does Clozapine cause weight gain? 2013. Available from: <https://www.hra.nhs.uk/planning-and-improving-research/application-summaries/research-summaries/why-does-clozapine-cause-weight-gain/>.

20. Leucht S, Arbter D, Engel RR, Kissling W, Davis JM. How effective are second-generation antipsychotic drugs? A meta-analysis of placebo-controlled trials. Molecular psychiatry. 2009;14(4):429-47.

21. Almond S, Knapp M, Francois C, Toumi M, Brugha T. Relapse in schizophrenia: costs, clinical outcomes and quality of life. The British Journal of Psychiatry. 2004;184(4):346-51.

22. National Collaborating Centre for Mental Health. Psychosis and Schizophrenia in Adults: The NICE Guideline on Treatment and Management. 2014.

23. Curtis L, Burns A. Unit Costs of Health and Social Care 2020. Caterbury: 2020. Available from: <https://www.pssru.ac.uk/project-pages/unit-costs/unit-costs-2020/>.

24. National Health Service (NHS). National Schedule of NHS Costs 2018/19. 2021.

25. NHS Digital. Hospital Episode Statistics for Admitted Patient Care and Outpatient Data. 2021. Available from: <https://digital.nhs.uk/data-and-information/publications/statistical/hospital-episode-statistics-for-admitted-patient-care-outpatient-and-accident-and-emergency-data>.

26. Curtis L, Burns A. Unit Costs of Health and Social Care. 2007.

27. Curtis L, Burns A. Unit Costs of Health and Social Care. 2017.

28. National Institute for Health and Care Excellence (NICE). Olanzapine. [Internet]. Available from: <https://bnf.nice.org.uk/drug/olanzapine.html#indicationsAndDoses>.

29. Department of Health and Social Care. Drugs and pharmaceutical electronic market information tool (eMIT). 2021. Available from: <https://www.gov.uk/government/publications/drugs-and-pharmaceutical-electronic-market-information-emit>.

30. Curtis L, Burns A. Unit Costs of Health and Social Care 2020. Canterbury: Personal Social Services Research Unit, University of Kent; 2020.

31. National Collaborating Centre for Mental Health. Psychosis and Schizophrenia in Adults: The NICE Guideline on Treatment and Management. 2014.

32. National Institute for Health and Care Excellence. Psychosis and schizophrenia in children and young people: recognition and management (CG155). London; 2013.

33. National Health Service (NHS). Drug Tariff 2021. [Internet]. 2021. Available from: <https://www.nhsbsa.nhs.uk/pharmacies-gp-practices-and-appliance-contractors/drug-tariff>.
